# Editorial: Immune cell development and differentiation in liver diseases

**DOI:** 10.3389/fcell.2024.1454495

**Published:** 2024-08-21

**Authors:** Xiaojing Wu, Antonios Chatzigeorgiou, Ying Shi, Liuluan Zhu

**Affiliations:** ^1^ Department of Hepatology, The First Hospital of Jilin University, Changchun, China; ^2^ Beijing Key Laboratory of Emerging Infectious Diseases, Institute of Infectious Diseases, Beijing Ditan Hospital, Capital Medical University, Beijing, China; ^3^ Beijing Institute of Infectious Diseases, Beijing, China; ^4^ Department of Physiology, Medical School, National and Kapodistrian University of Athens, Athens, Greece

**Keywords:** hepatitis, liver fibrosis, liver cancer, liver transplantation, immune cell, development and differentiation

## 1 Introduction

Liver is a vital organ that acts as a reservoir for immune cells and is central to immune responses. The pathophysiological microenvironment of the liver provides an important site for the development and differentiation of immune cells such as T lymphocytes, natural killer (NK) cells and NKT cells, macrophages and dendritic cells (DCs). Macrophages are important immune cells in innate immunity, and have remarkable heterogeneity and polarization. Under pathological conditions, in addition to the resident macrophages, circulating monocytes are recruited to the diseased tissues, differentiate into macrophages and polarize to various phenotypes, mainly classically activated M1 and alternatively activated M2. Hepatitis is inflammatory condition of the liver caused by viruses, toxicants, alcohol or metabolic dysfunction. In various types of hepatitis, polarization of macrophages leads to a positive feedback loop of inflammation and tissue damage (Fernández-Regueras et al., 2023). T cell activation and differentiation are regulated in the presence of antigens in naïve T cells, potentially leading to T cell dysfunction, including exhaustion or senescence, and effect-mediated immune deficiency, which is important for host defense. The senescence and exhaustion of T cells are key instigators of chronic inflammation. During the progression from hepatitis to hepatocellular carcinoma, the exhaustion of CD4^+^ and CD8^+^ T cells, along with an increased proportion of regulatory T cells (Tregs), promotes tumor cell growth (Wang et al., 2021). In line with DCs in other tissues, hepatic DCs can also be classified in two distinct categories: conventional DCs (cDCs) and plasmacytoid DCs (pDCs). CD1d-restricted NKT cells are classified into two main subsets: type I or invariant NKT cells and type II or diverse NKT cells. In the context of liver transplantation rejection, different subsets of DCs and NKT cells exhibit dual roles in promoting and inhibiting inflammatory responses (Du et al.; Zhao et al.). Despite the significant attention that the role of various immune cells in liver diseases has received in scientific literature, there is still uncertainty about the mechanisms that regulate the abnormal development and differentiation of these cells within the liver. The factors that influence the trajectory of immune cell development in the liver are not yet fully understood. This editorial aims to provide a comprehensive overview of the development and differentiation of immune cells in the context of liver diseases.

In this Research Topic, we have gathered four insightful papers that delve into the intricate relationship between immune cell development and various liver diseases. We extend our heartfelt thanks to the authors, reviewers, and our dedicated editorial team for their invaluable contributions to advancing our understanding of this critical field.

## 2 Development and differentiation of immune cells in hepatitis

While the use of vaccines and antiviral drugs has reduced the incidence of hepatitis, the rise in metabolic disorders has led to an increase in alcohol-related and non-alcoholic fatty liver diseases. In this Research Topic, Papadopoulos et al. elucidated how immune cells differentiate and develop in the context of these metabolic disorders, highlighting the role of abnormal oxidation in triggering inflammation and tissue damage. It also discusses the early stages of alcoholic liver disease, where similar processes of lipid degeneration and damage occur, and the subsequent activation of immune responses (Papadopoulos et al.).

## 3 Development and differentiation of immune cells in liver fibrosis and liver cancer

Liver fibrosis and cancer occur as a consequence of various liver diseases, during which significant alterations take place in the development and differentiation of immune cells. Throughout the course of alcoholic liver disease (ALD) progression, repeated episodes of inflammation resolution and tissue repair lead to an increased polarization of M2 macrophages (Sun et al.). Prolonged activation of M2 macrophages can induce pathological fibrosis due to their role in promoting tissue repair. Furthermore, M2 macrophages interact with hepatic stellate cells (HSCs) to establish a positive feedback loop that enhances collagen deposition (Cao et al., 2022). During the transition from liver disease to hepatocellular carcinoma (HCC), diverse phenotypic changes in immune cells collectively contribute to the formation of the tumor microenvironment. For instance, tumor-associated macrophages (TAMs) predominantly express an immunosuppressive M2 phenotype, which promotes tumor development, growth, dissemination, and stimulates neovascularization (Cao et al., 2024). Additionally, various T cell subsets such as CD4^+^ and CD8^+^ T cells are depleted in HCC leading to weakened immune surveillance function and insufficient anti-tumor immune response (Blank et al., 2019). Regulatory T cells exacerbate this process (Wang et al., 2021). Although there is some controversy surrounding it, innate killer T (iNKT) and mucin-associated invariant T (MAIT) cell subsets may have either promoting or inhibitory roles in HCC progression (Papanastasatou and Verykokakis, 2023).

## 4 Development and differentiation of immune cells in liver transplantation

Liver transplantation is the most effective approach for managing end-stage liver diseases. However, complications such as ischemia-reperfusion injury (IRI), rejection, tolerance, and graft infections are closely associated with immune cell differentiation and subpopulations. Two review articles in this edition provide a comprehensive overview of these subjects (Zhao et al.; Du et al.). Liver dendritic cells are less mature than those in peripheral tissues, with lower levels of MHC-II and other molecules that typically activate T cells. They contribute to immune tolerance by producing anti-inflammatory factors like prostaglandin E2, IL-10, and indoleamine 2,3-dioxygenase (IDO), which help to suppress the immune response to a transplant (Ness et al., 2021; Du et al.). NKT cells have a dual role post-transplant. While some activated NKT cells can trigger liver cell death and promote rejection, others help to regulate the immune response and foster tolerance, potentially enhancing the survival of the transplanted liver. The balance between these NKT cell subsets and their inflammatory or regulatory functions is crucial for the success of liver transplantation (Jukes et al., 2012; Jukes et al., 2007).

## 5 Therapeutic strategies and prospects for immune cell development and differentiation in liver diseases

As previously discussed, the development and differentiation of immune cells play a crucial role in the progression and inhibition of various types of liver diseases. Numerous immunotherapies have emerged, with the central aim being the restoration and maintenance of immune balance. For instance, mediators and drugs that modulate macrophage activation and polarization, such as berberine, aspirin, and metformin, have been shown to attenuate M1 polarization and reduce excessive liver tissue damage during hepatitis (Li et al., 2019; Oh et al., 2019; Patel et al., 2021). Similarly, in liver transplantation, adoptive transfer of regulatory immune cells, such as tolerogenic dendritic cells (Tol-DC), can promote donor-specific tolerance and reduce the need for immunosuppressants (Willekens et al., 2021). Conversely, in hepatocellular carcinoma, immune checkpoint inhibitors (ICIs) are utilized to counteract tumor-induced immunosuppression, thereby activating CD4^+^ and CD8^+^ cells to target and destroy tumor cells (Agdashian et al., 2019).

## 6 Summary

We are honored to present this Research Topic of research, which provides a thorough analysis of how immune cells develop and differentiate in different liver diseases and the therapeutic opportunities this presents. Covering a range of prevalent liver diseases, these articles shed light on the underlying mechanisms and explore the potential of immunotherapy as a treatment approach.
